# Redo pancreaticojejunal anastomosis for late-onset complete pancreaticocutaneous fistula after pancreaticojejunostomy

**DOI:** 10.1186/s12957-022-02687-y

**Published:** 2022-07-04

**Authors:** Michihiro Yamamoto, Masazumi Zaima, Tekefumi Yazawa, Hidekazu Yamamoto, Hideki Harada, Masahiro Yamada, Masaki Tani

**Affiliations:** Department of Surgery, Shiga General Hospital, 4-30 Moriyama 5-chomeShiga Prefecture, Moriyama City, 524-8524 Japan

**Keywords:** Pancreaticoduodenectomy, Pancreatic fistula, Pancreaticojejunostomy

## Abstract

**Background:**

Pancreaticojejunal (PJ) anastomosis occasionally fails several months after pancreaticoduodenectomy (PD) with Child reconstruction and can ultimately result in a late-onset complete pancreaticocutaneous fistula (Lc-PF). Since the remnant pancreas is an isolated segment, surgical intervention is necessary to create internal drainage for the pancreatic juice; however, surgery at the previous PJ anastomosis site is technically challenging even for experienced surgeons. Here we describe a simple surgical procedure for Lc-PF, termed redo PJ anastomosis, which was developed at our facility.

**Methods:**

Between January 2008 and December 2020, six consecutive patients with Lc-PF after PD underwent a redo PJ anastomosis, and the short- and long-term clinical outcomes have been evaluated.

The abdominal cavity is carefully dissected through a 10-cm midline skin incision, and the PJ anastomosis site is identified using a percutaneous drain through the fistula tract as a guide, along with the main pancreatic duct (MPD) stump on the pancreatic stump. Next, the pancreatic stump is deliberately immobilized from the dorsal plane to prevent injury to the underlying major vessels. After fixing a stent tube to both the MPD and the Roux-limb using two-sided purse-string sutures, the redo PJ anastomosis is completed using single-layer interrupted sutures. Full-thickness pancreatic sutures are deliberately avoided by passing the needle through only two-thirds of the anterior side of the pancreatic stump.

**Results:**

The redo PJ anastomosis was performed without any intraoperative complications in all cases. The median intraoperative bleeding and operative time were 71 (range 10–137) mL and 123 (range 56–175) min, respectively. Even though a new mild pancreatic fistula developed postoperatively in all cases, it could be conservatively treated within 3 weeks, and no other postoperative complications were recorded. During the median follow-up period of 92 (range 12–112) months, no complications at the redo PJ anastomosis site were observed.

**Conclusions:**

This research shows that the redo PJ anastomosis for Lc-PF we developed is a safe, feasible, and technically no demanding procedure with acceptable short- and long-term clinical outcomes. This procedure has the potential to become the preferred treatment strategy for Lc-PF after PD.

## Background

Late-onset complete pancreaticocutaneous fistula (Lc-PF) can occur several months after pancreaticoduodenectomy (PD) with Child reconstruction and is thought to be due to pancreatic juice pooling at the anastomosis site, resulting in a pseudocyst; subsequent pseudocyst enlargement causes failure of the PJ anastomosis. Eventually, an Lc-PF develops because the placement of a percutaneous drain into the pseudocyst allows the pancreatic juice to entirely discharge out through the fistula tract. Since the main pancreatic duct (MPD) of the remnant pancreas does not communicate with the gastrointestinal tract, creating internal drainage for the pancreatic juice is necessary for treating the fistula [1–6].

Direct surgical management of such failure of the PJ anastomosis is considered technically demanding because intraabdominal dissection in the chronic phase after PD is unsafe due to the presence of tight adhesions, scar tissue, obscure anatomy, and risk of critical injury to adjacent major vessels or organs, even if performed by experienced pancreatic surgeons [1, 7–9]. Although alternative radiologic and endoscopic management strategies have been reported as small case series [10–14], these nonsurgical treatments are not yet the standard procedure [15] due to large and patient-dependent variations in peri-pancreatic anatomy after PD.

To overcome these problems, we have developed a simple surgical procedure for Lc-PF after PD and have safely performed it on six consecutive patients. Here, we describe the procedure and retrospectively review its safety, feasibility, and short- and long-term clinical outcomes.

## Methods

Data in the pancreatic resection database of our institution were used after the approval of the institutional board (approval number 180921).

Between January 2007 and December 2020, 293 patients underwent PD with Child reconstruction at our institution; among them, 6 patients (2.0%) developed Lc-PF who all underwent the redo PJ anastomosis. Primary conditions requiring PD are listed in Table 1.

**Table 1:** Clinical findings of patients who underwent redo PJ-anastomosis for late-onset complete pancreaticocutaneous fistula after pancreaticoduoenectomy

Case	Age/sex	Primary disease	Condition of the pancreas at PD	Between PD and Lc-PF (months)	Pseudo-cyst size before puncture (mm)	Between Lc-PF formation and redo PJ anastmosis (days)	Amylase level of discharge (IU/mL)	Amount of discharge (mL/day)	Operative time (min.)	Intra- operative blood loss (mL)	Post- operative hospitali-zation (days)	Post-operative complication	Follow-up duration (months)	Compli- cation at redo PJ site
1	76/F	IPMA	Soft	6	66	7	57,000	300	175	81	11	PF (grade A)	109	No
2	80/F	BDC	Soft	2	42	4	40,000	110	137	125	20	PF (grade A)	12	No
3	56/F	GBC	Soft	1	37	3	106,600	280	115	61	21	PF (grade A)	112	No
4	71/M	Pancreatic NET	Soft	9	63	11	NM	NM	130	137	14	PF (grade A)	105	No
5	67/M	Duodenal cancer	Soft	3	30	37	NM	NM	91	10	27	PF (grade A)	60	No
6	60/M	GIST	Soft	3	26	60	NM	200	56	13	41	PF (grade A)	79	No

*PD* pancreaticoduodenectomy, *Lc-PF* late-onset complete pancreaticocutaneous fistula, *PJ* pancreaticojejunal, *IPMA* intraductal papillary-mucinous adenoma, *BDC* bile duct cancer, *GBC* gall bladder cancer, *NET* neuroendocrine tumor, *GIST* gastrointestinal stromal tumor, *PF* pancreatic fistula, *NM* not measured accurately

The PJ anastomosis at PD was performed in all 293 patients using the Kakita procedure [16], one of the most widely accepted procedures for PJ in Japan [17]. For patients with a soft pancreas, an external pancreatic drainage tube was added. For patients with a hard pancreas, an internal pancreatic tube was placed as a lost stent. Postoperative somatostatin analogs were not routinely administered.

**Fig. 1:**
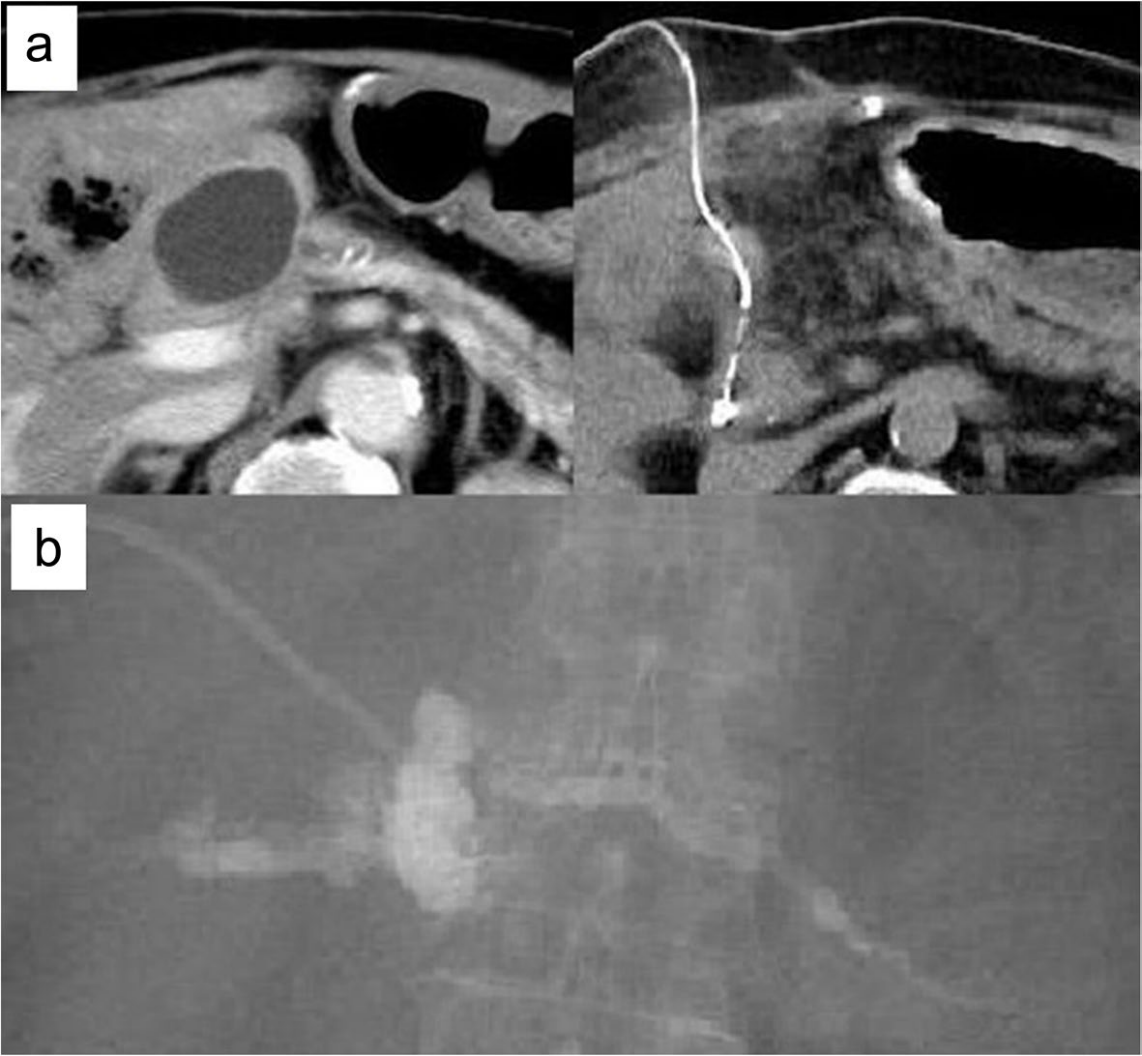
Preoperative computed tomography (CT) and percutaneous fistulography findings. **a** CT indicates failure of the pancreaticojejunal anastomosis with pseudocyst formation, including pooled pancreatic juice and dilation of the main pancreatic duct (MPD) of the remnant pancreas. **b** The percutaneous fistulography shows MPD that does not communicate with the Roux-limb

Lc-PF typically presented as upper abdominal pain with computed tomography (CT) revealing the failure of the PJ anastomosis with pseudocyst formation and MPD dilation of the remnant pancreas (Fig. 1a). Therefore, the pancreatic juice was entirely discharged out through the fistula created by a percutaneous puncture and drain placement. Percutaneous fistulography was used to visualize the dilated MPD without the Roux-limb as they no longer communicate with each other (Fig. 1b), and Lc-PF was diagnosed based on these findings. The peri-pancreatic anatomy was evaluated in detail using three-dimensional CT, fistulography, and ultrasonography data, and no critical recurrences of the primary malignant disease were detected in all patients. The inflammatory reaction after PD already disappeared, and the patient has sufficient nutrition; thus, the patient’s physical condition was categorized as favorable. Written informed consent for the redo PJ anastomosis was obtained from all patients, and the procedure was attempted as soon as possible after the Lc-PF diagnosis under the situation that the patient is in good general condition.

### Surgical procedure

**Fig. 2:**
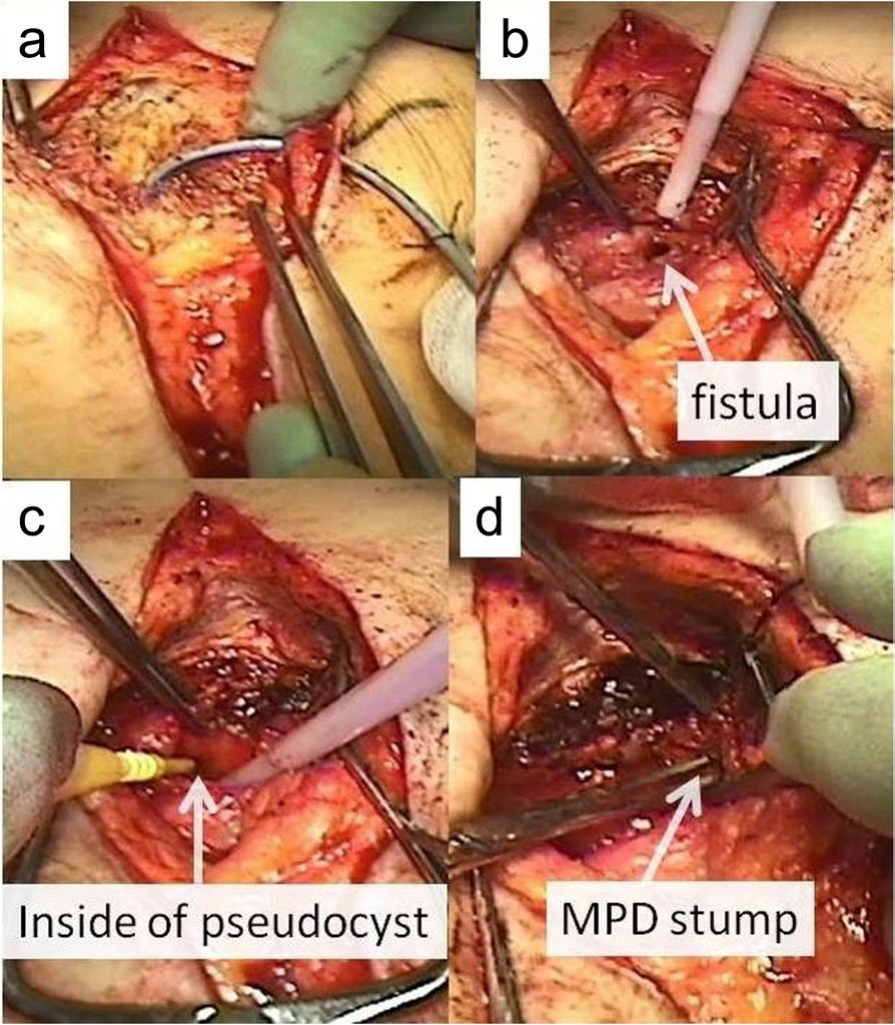
Surgical procedure to the previous PJ anastomosis site. **a** An approximately 10-cm midline incision just above the previous pancreaticojejunal (PJ) anastomosis site is created. **b** Intra-abdominal cavity is carefully and minimally dissected, as required, toward the previous PJ anastomosis site using a drain placed in the fistula tract as a guide. **c** Pseudocyst is situated between the pancreatic stump and Roux-limb. **d** The stump of the main pancreatic duct is detected within the pseudocyst

An approximately 10-cm midline skin incision was created just above the primary PJ anastomosis site, which was identified using CT or ultrasonography imaging. The upper abdominal cavity was carefully and minimally dissected using the drain placed through the fistula tract that acted as the guide (Fig. 2a, b). After the failed PJ anastomosis was reached (Fig. 2c), the MPD stump was identified on the pancreatic stump within the pseudocyst (Fig. 2d). The MPD and pancreatic stump were found to be more dilated and harder than at the previous PD. When identifying the MPD stump was difficult due to the presence of thick connective tissues, as seen in case 4 in this series, slicing the pancreatic stump under intraoperative ultrasonographic guidance exposed the MPD. The pancreatic stump was intentionally immobilized from the dorsal plane because it is technically demanding and unsafe to separate it from the strongly adhering superior mesenteric vein (SMV), splenic vein (Sp.V), and common hepatic artery (CHA).

**Fig. 3:**
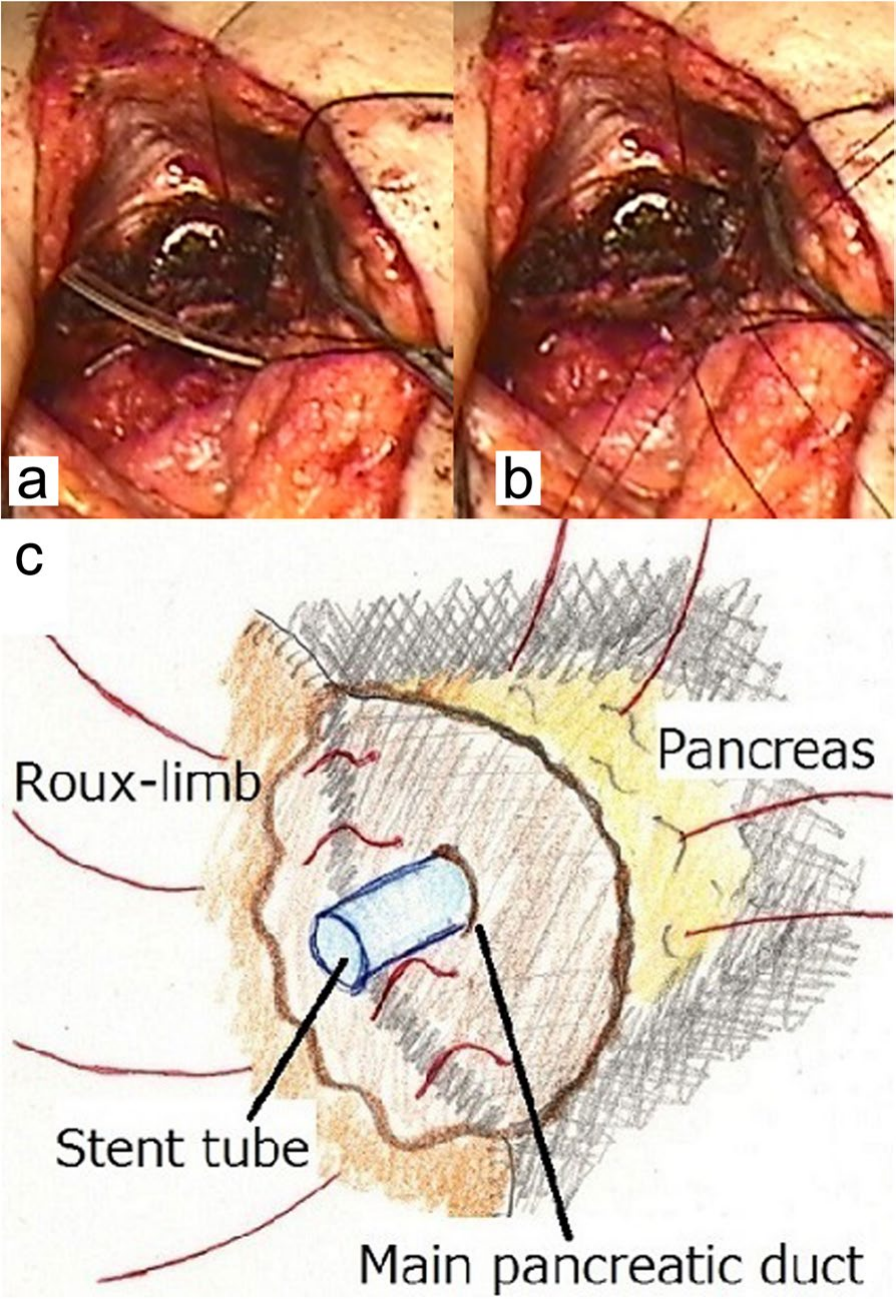
Surgical procedure of the redo PJ anastomosis**. a** A 8-Fr pancreatic tube, trimmed approximately 3- cm in length, is placed within the main pancreatic duct (MPD) as a lost stent. **b** The pancreatic stump and Roux-limb are re-anastomosed end-to-side using 3–0 nonabsorbable single-layer interrupted sutures. **c** Every anastomotic suture passes through only two-thirds of the anterior side of the pancreatic stump instead of the full-thickness sutures as in the Blumgart anastomosis

The previous Roux-limb could be easily identified because it is located opposite the pancreatic stump. As the wall is flexible and nonfragile, which is in contrast to that observed in the acute phase after PD, there is no need to newly create another Roux-limb. An 8-Fr pancreatic tube, trimmed to approximately 3 cm in length, was placed into both sides as a lost stent (Fig. 3a) and fixed using two-sided purse-string sutures, allowing the pancreatic juice to totally drain into the Roux-limb through the stent. The pancreatic stump and Roux-limb were re-anastomosed end-to-side using 3–0 nonabsorbable single-layer interrupted sutures (Fig. 3b). To prevent injury to the adhered major vessels or contiguous organs, every anastomotic suture passed through only two-thirds of the anterior side of the pancreatic stump (Fig. 3c), that is, full-thickness sutures used in the Blumgart anastomosis [18, 19] were deliberately avoided. The minute bite of these pancreatic stitches is not concerning because the pancreatic stump less likely tears at the ligation due to its hard texture and the fact that it is covered with thick connective tissues.

A peritoneal drain was placed around the PJ anastomosis; however, a feeding jejunostomy for enteric nutrition was not needed, and neither of the postoperative somatostatin analogs were necessary.

## Results

The redo PJ anastomosis was successfully performed in all six patients without any intraoperative complications, including injuries to the adjacent organs or vessels. Table 1 shows the preoperative clinical characteristics and operative results. All six patients with Lc-PF had a soft pancreas and no pancreatic cancer at PD (Table 1). The median values for intraoperative bleeding and operative time were 71 (range 10–137) mL and 123 (range 56–175) min, respectively. Postoperative grade A pancreatic fistula (PF), as defined by the International Study Group for Pancreatic Fistula classification [20, 21], occurred and could be conservatively treated within 3 weeks in all six patients. Other postoperative complications were not recorded in any patient. The median length of postoperative hospital stay was 20 (range 11–41) days. During the median follow-up period of 92 (range 12–112) months, no complications in the redo PJ anastomosis site or PF recurrence were observed. Currently, four patients are alive and remain to be primary disease-free, whereas the other two patients died of cancer recurrence.

## Discussion

The redo PJ anastomosis for Lc-PF after PD described here appears to be both safe and feasible with acceptable short- and long-term clinical outcomes.

In Lc-PF, completion of pancreatectomy or internal drainage of the pancreatic juice into the gastrointestinal tract is required to treat the fistula because the remnant pancreas is an isolated segment and the entire pancreatic juice drains out only through the percutaneous fistula. However, completion of pancreatectomy is not recommended due to substantial postoperative metabolic abnormalities, including severe brittle diabetes [22–24]. Thus, internal drainage is considered ideal for Lc-PF management.

Recently, a few small numbers of case studies have reported successful radiologic or endoscopic interventions for establishing internal drainage [10–14]; however, these nonsurgical strategies may not become the standard treatment because of the enormous variation in peri-pancreatic anatomy of each patient [15]. Conversely, direct surgical management of the previous PJ site after PD in such patients is not routinely performed because it is considered technically challenging even for experienced pancreatic surgeons [7, 12]. To the best of our knowledge, no reports have yet described redo PJ anastomosis for Lc-PF. Here, we have described and developed a simple surgical procedure for Lc-PF after PD and showed that it can be safely performed.

This redo PJ anastomosis has the following four technical advantages. First, the percutaneous drain through the fistula tract can be used as a guide identifying the previous PJ anastomosis site. This tube allows secure assessment and minimal range of intraabdominal dissection even through a small skin incision. Surrounding adhesions are intentionally left untouched to prevent or limit postoperative inflammation due to PF, bacterial infection, or other causes. Thus, the previous PJ anastomosis site could be easily accessed using this method in all our patients, and importantly, neither postoperative intraabdominal abscess formation nor extensive inflammation was observed despite new PF formation occurring in all patients. Second, the adjacent major vessels and organs remain covered and protected by chronic connective scar tissues, a finding in contrast to that observed in the acute phase after PD [25, 26]. These connective tissues reduce the risk of critical injury to the adjacent vessels and organs during intraabdominal dissection. Further, we found that the original Roux-limb wall remained firm because of those presence of connective tissues and that it tolerated the redo anastomosis, thereby eliminating the need to create another one. Third, the redo PJ anastomosis utilizes an observation that pancreatic texture is hard with the ends of all micro-pancreatic-ducts on the pancreatic stump occluded by the connective tissues; this was conclusively established by preoperative fisulography showing no leakage of the contrast media. Therefore, we assumed that the entire surface of the pancreatic stump had not been necessarily encapsulated by the Roux-limb wall, i.e., that every anastomotic suture only needed to predominantly pass through the anterior side of the pancreatic stump (Fig. 3c) instead of the full-thickness sutures as utilized in the Blumgart anastomosis [18, 19]. Consequently, the technically unsafe procedure of the previous pancreatic stump mobilization from the underlying and adhered SMV, Sp.V, and CHA is not required. Moreover, this hard texture of the pancreas makes it less likely to tear at ligature sites even if every suture is not full-thickness. Fourth, in contrast to PF in the acute phase after PD, which can be complicated by sepsis, intraabdominal abscess, ruptured pseudoaneurysm, or low nutrition status, the physical condition of patient who underwent redo PJ anastomosis remains favorable [1]. We believe that these four advantages contributed to the acceptable outcomes reported in our study.

Currently, the duct-to-mucosa PJ anastomosis is increasingly considered a standard procedure during PD [27]; however, we did not use this technique for the Lc-PF because the technical advantage and simplicity of the redo procedure should be retained by making a small abdominal incision. The current duct-to-mucosa PJ anastomosis is technically more complicated and requires a sufficiently large surgical field; therefore, it was found unsuitable for Lc-PF.

Fistuloenterostomy has been reported as an effective management strategy for internal drainage in refractory and persistent PF after PD [28, 29], which could also be a surgical option for Lc-PF. Such a procedure would not require identification of the previous PJ anastomosis site, which is in contrast to our technique. However, this procedure has two disadvantages compared to the redo PJ anastomosis described here. First, a 4-month or longer waiting period is required so that the fistula becomes sufficiently hard to tolerate the anastomosis after drain placement. Second, the fistula tract carries a potential risk of postoperative stenosis or necrosis due to the lack of blood supply [30]. Conversely, our redo PJ anastomosis does not have a waiting period or carry ischemic risk, thereby making it more feasible than the fistuloenterostomy for Lc-PF management.

Despite these advantages, this study still has some limitations. First, this study comprised a small number of patients, which is due to the low incidence of Lc-PF. Further, since this was a single-center study, identifying eligible patients was also difficult. Thus, future studies must collect clinical data from multiple centers. Second, all six patients had no pancreatic cancer at PD (Table 1). For patients with pancreatic cancer, the initiation or continuation of postoperative chemotherapy would be prevented by redo PJ anastomosis, possibly leading to a negative impact on patient survival. Although all patients in our study were treated within 3 weeks postoperatively, redo PJ anastomosis should be carefully performed on patients with pancreatic cancer, in particular those with recurrent cancer. Third, the postoperative hospitalization was long but could be related to the Japanese insurance system wherein hospitalization cost is relatively low.

## Conclusions

The redo PJ anastomosis for Lc-PF after PD described here is both safe and feasible with acceptable short- and long-term clinical outcomes. Further, we believe that the redo PJ anastomosis is not as demanding as expected; thus, this procedure may become the preferred management option for Lc-PF after PD. 

## Data Availability

Datasets used or analyzed during the current study are available from the corresponding author on reasonable request.

## Abbreviations

PJ: Pancreaticojejunal
PD: Pancreaticoduodenectomy
Lc-PF: Late-onset complete pancreaticocutaneous fistula
MPD: Main pancreatic duct
CT: Computed tomography
SMV: Superior mesenteric vein
Sp.V: Splenic vein
CHA: Common hepatic artery
PF: Pancreatic fistula
